# Breast cancer and human papillomavirus infection: No evidence of HPV etiology of breast cancer in Indian women

**DOI:** 10.1186/1471-2407-11-27

**Published:** 2011-01-20

**Authors:** Suresh Hedau, Umesh Kumar, Showket Hussain, Shirish Shukla, Shailja Pande, Neeraj Jain, Abhishek Tyagi, Trivikram Deshpande, Dilafroze Bhat, Mohammad Muzaffar Mir, Sekhar Chakraborty, Y Mohan Singh, Rakesh Kumar, Kumaravel Somasundaram, Alok C Bharti, Bhudev C Das

**Affiliations:** 1 Division of Molecular Oncology, Institute of Cytology & Preventive Oncology (ICMR), I-7, Sector - 39, Noida - 201 301, India; 2 Dr. B.R. Ambedkar Centre for Biomedical Research, University of Delhi, Delhi - 110 007, India; 3 Department of Zoology, Goa University, Goa - 403206, India; 4 Department of Clinical Biochemistry, Sher-I-Kashmir Institute of Medical Sciences, Soura, Srinagar, Jammu and Kashmir, India; 5 College of Medicine, Al-Souf University Sakaka, Kingdom of Saudi Arabia; 6 Department of Pathology, Silchar Medical College, Silchar-788014, India; 7 Department of Pathology, Regional Institute of Medical Science, Imphal - 795004, India; 8 Department of Microbiology & Cell Biology, Indian Institute of Science, Bangalore - 560012, India

## Abstract

**Background:**

Two clinically relevant high-risk HPV (HR-HPV) types 16 and 18 are etiologically associated with the development of cervical carcinoma and are also reported to be present in many other carcinomas in extra-genital organ sites. Presence of HPV has been reported in breast carcinoma which is the second most common cancer in India and is showing a fast rising trend in urban population. The two early genes E6 and E7 of HPV type 16 have been shown to immortalize breast epithelial cells in vitro, but the role of HPV infection in breast carcinogenesis is highly controversial. Present study has therefore been undertaken to analyze the prevalence of HPV infection in both breast cancer tissues and blood samples from a large number of Indian women with breast cancer from different geographic regions.

**Methods:**

The presence of all mucosal HPVs and the most common high-risk HPV types 16 and 18 DNA was detected by two different PCR methods - (i) conventional PCR assays using consensus primers (MY09/11, or GP5+/GP6+) or HPV16 E6/E7 primers and (ii) highly sensitive Real-Time PCR. A total of 228 biopsies and corresponding 142 blood samples collected prospectively from 252 patients from four different regions of India with significant socio-cultural, ethnic and demographic variations were tested.

**Results:**

All biopsies and blood samples of breast cancer patients tested by PCR methods did not show positivity for HPV DNA sequences in conventional PCRs either by MY09/11 or by GP5+/GP6+/HPV16 E6/E7 primers. Further testing of these samples by real time PCR also failed to detect HPV DNA sequences.

**Conclusions:**

Lack of detection of HPV DNA either in the tumor or in the blood DNA of breast cancer patients by both conventional and real time PCR does not support a role of genital HPV in the pathogenesis of breast cancer in Indian women.

## Background

Breast cancer is the second most common cancer in the world, after the cancer of the lung, affecting one in eight women during their lifetime, but it is the leading cancer among women worldwide [1]. In India breast cancer is the second most dominant cancer in women, but it is showing a fast rising trend in major metropolitan cities in India [2]. Although various clinico-epidemiologic, genetic and epigenetic factors including mutations in breast cancer susceptibility genes *BRCA1 *and *BRCA2 *[3,4], sex-steroid hormones and lifestyle factors have been strongly implicated in the development of breast cancer, the mechanism(s) of breast carcinogenesis is still not clearly understood.

High-risk human papillomaviruses (HR-HPVs) are carcinogenic viruses which are primarily associated with cervical cancer but are also linked with other anogenital cancers and cancers of other organ sites [5] such as oral cavity [6-8], esophagus [9-11], nasopharyngeal and laryngeal carcinoma [5,12-15] and possibly in retinoblastoma [16-18]. In India, HPV is found to be present in 100% of cervical cancer specimens and prevalence of high risk HPV type 16 is exceptionally high (~90%) in them [19]. The HR-HPV oncoproteins E6 and E7 which have been found to interact and inactivate the two principal host cell tumor suppressor proteins p53 and Rb respectively [20-22] are also shown to immortalize human mammary epithelial cells in-vitro [23,24]. Several other viruses have also been implicated in the etiology of human breast cancer [23,24] but these are not confirmed by other authors [25,26].

Reports on the distribution of HPV infection in breast cancer are not only limited but also highly controversial. Several authors [27-31] including our previous report on fine needle aspirated breast cancer cells [32] did not find any HPV infection in breast cancer. A moderate frequency of 20-48% HPV infection was reported by many authors [33-42], whereas a very high frequency of HPV infection ranging from 60 to 85% occurrence of HPV in breast cancer was reported by others [43-46]. Most interesting is recent demonstration of high risk HPV18 in the breast cancer cell lines by in-situ hybridization and observation of HPV-specific koilocytes in breast cancer cells which reiterates the oncogenic role of HPV in breast cancer [47]. This prompted us to re-look into the role of HPV in a large number of breast cancer cases that were previously analyzed for mutations in BRCA1, BRCA2 and p53 tumor suppressor genes [48] and collected from all four different regions of India. We used both conventional and real time PCR assays with consensus (MY09/11, GP5+/GP6+) and type specific (HPV16 E6/E7) primers to detect HPV DNA sequences in tumors as well as corresponding blood samples of same breast cancer patients. The results demonstrate complete absence of HPV DNA in breast cancer patients in India.

## Methods

### Breast cancer patient population and sample collection

A total of two hundred fifty two breast cancer patients (n = 252) were recruited for analysis of HPV infection from different geographic regions of India. The region wise distribution of samples is presented in Table 1. Biopsies and blood samples were collected directly from the surgical OT of respective hospitals (listed in Table 1) in chilled phosphate-buffered saline and stored at -70º C till further processing. 24 blood samples of familial breast cancer cases collected (without biopsies) from Goa, a distinct geographic region of India. The majority of the cases were classified as infiltrating ductal carcinoma. Written informed consent was obtained from all the subjects included in the study and clinico-epidemiological details were taken from their clinical records. The study was approved by Institutional Ethics Committee (IEC) of all the collaborating Institutes and hospitals - Lok Nayak Hospital, New Delhi; Sher-I-Kashmir Institute of Medical Sciences, Srinagar, Jammu & Kashmir; Regional Institute of Medical Science, Imphal; Assam Medical College, Dibrugarh; Silchar Medical College, Silchar; Goa Medical College, Goa and Kidwai Memorial Institute of Oncology, Bangalore in accordance with the guidelines of Indian Council of Medical Research (ICMR) and Helsinki Declaration. None of the breast cancer patients had history of any other cancer or HPV infection and all were inhabitants of India. The age of the breast cancer patients recruited varied from 25-80 years, the mean age being 51.5 ± 16.7.

**Table 1:** Distribution of breast biopsies and blood samples from the different regions of India

Name of the Hospitals	Biopsies	Blood	Matched biopsy & blood
Lok Nayak Hospital, New Delhi	100	-	-
Sher-I-Kashmir Institute of Medical Sciences, Srinagar, Jammu & Kashmir	43	43	43
Regional Institute of Medical Science, Imphal Assam Medical College, Dibrugarh Silchar Medical College, Silchar	65	65	65
Goa Medical College, Goa	-	24	-
Kidwai Memorial Institute of Oncology, Bangalore	20	10	10
**Total**	**228**	**142**	**118**

### DNA extraction for PCR

**Figure 1:**
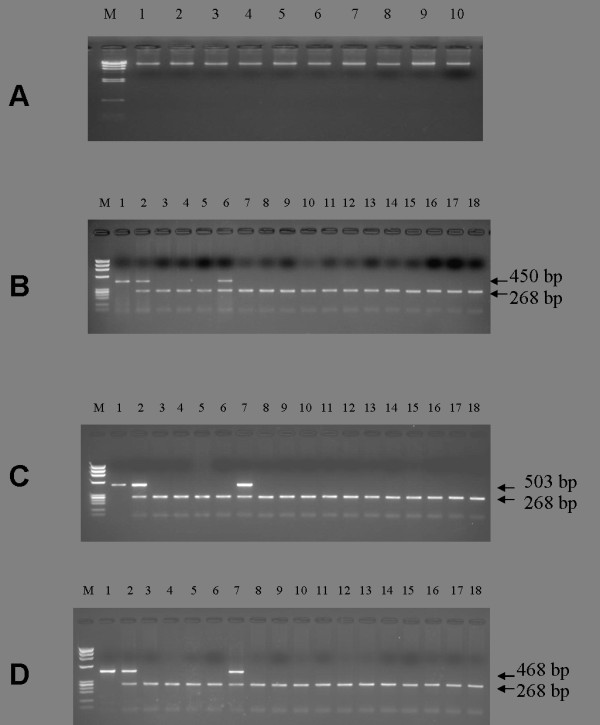
**(A-D) Quantity and quality of genomic DNA from breast cancer biopsy and PCR amplification of different regions of HPV genome**. **A**: Estimation of quantity and quality of phenol-chloroform extracted genomic DNA from breast cancer biopsies as visualized on an ethidium bromide-stained 1% agarose gel. Lane M: Hind III-digested λ-DNA molecular weight marker. Lane 1-10: Genomic DNA samples from breast cancer biopsies. **B**: PCR amplification of HPV L-1 consensus sequences showing the amplimer of 450 bp along with β-globin of 268 bp. Lane M: Hae III-digested φ × 174 DNA molecular weight marker. Lane 1: positive control (HPV16 plasmid DNA), Lane 2: HPV16 positive cervical cancer biopsy DNA, Lane 3: Blood DNA from breast cancer patient, Lane 4: HPV negative cell line (C33a) DNA, Lane 5: MCF-7 cell DNA, Lane 6: HeLa cell DNA, Lanes 7-18: breast cancer biopsy DNA. All were positive for β-globin but none of them positive for HPV. **C**: PCR amplification of HPV16 E6 showing the amplimer of 503 bp along with β-globin of 268 bp. Lane M: Hae III-digested φ × 174 DNA molecular weight marker. Lane 1: positive control (HPV16 plasmid DNA), Lane 2: Cervical cancer DNA, Lane 3: Blood DNA from breast cancer patient, Lane 4: HPV negative cell line (C33a) DNA, Lane 5: MCF-7 cell DNA, Lane 6: Human placental DNA, Lane 7:SiHa cell DNA, Lanes 8-18: breast cancer biopsy DNA showing positivity for β-globin but negative for HPV. **D**: PCR amplification of HPV16 E7 showing the amplimer of 468 bp along with β-globin of 268 bp. Lane M: Hae III-digested φ × 174 DNA molecular weight marker. Lane 1: positive control (HPV16 plasmid DNA), Lane 2: Cervical cancer DNA, Lane 3: Blood DNA from breast cancer patient, Lane 4: HPV negative cell line (C33a) DNA, Lane 5: MCF-7 cell DNA, Lane 6: Human placental DNA, Lane 7:SiHa cell line DNA, Lane 8-18: breast cancer biopsy DNA showing positivity for β-globin but negative for HPV.

Cellular DNA from freshly collected breast tumor biopsies and peripheral venus blood were isolated using standard Proteinase K digestion, phenol-chloroform extraction and ethanol precipitation method routinely being used in our laboratory [19,49,50]. The quality and concentration of DNA was measured either on an ethidium bromide-stained 1% agarose gel using Hind III-digested lambda marker (Figure 1A) or by standard spectrophotometric methods.

### Conventional PCR using L1 consensus MY09/11 and GP5+/GP6+ or HPV16 E6/E7 primers

Approximately, 100-200 ηg cellular DNA was utilized for conventional PCR using the protocol routinely followed in our laboratory [48,49,51]. For the detection of HPV DNA, both conventional as well as qRT-PCR methodology were employed using most common L1 consensus primers (MY09/11, GP5+/GP6+) or type-specific HPV16 E6/E7 primers. HPV16 plasmid DNA as well as HPV16 positive tumor DNA from cervical cancer patients or HeLa DNA served as positive controls whereas HPV negative cell line C33a DNA or human placental DNA served as negative control. Amplification of β- globin gene as well as exon 5 of p53 tumor suppressor gene served as internal controls to examine quality, integrity and successful amplification of breast tissue DNA. Sequences of consensus primers (MY09/MY11 and GP5+/GP6+) located within the conserved L1 region of HPV genome, HPV16 E6 and E7 primers along with β-globin and p53 of exon 5 primer sequences are presented in Table- 2.

**Table 2:** Oligonucleotide primer sequences used for the amplification of different regions of HPV and internal control.

*Primers*	*Primer Sequences*	*Location*	*Annealing* *temperature* *(ºC)*	*Amplimer size* *(bp)*	*Reference*
MY09	5'-GCM CAG GGW CAT AAY AAT GG-3'	L1	55	450	[49]
MY11	5'-CGT CCM ARR GGA WAC TGA TC-3' where M = A + C, W = A + T, Y = C + T, R = A + G).				
GP5+	5'-TTT GTT ACT GTG GTA GAT ACT AC-3'	L1	40	145	[51]
GP6+	5'-CTT ATA CTA AAT GTC AAA TAA AAA-3'				
HPV16 E6 (F)	5'-GAA ACC GGT TAG TAT AAA AGC AGA C-3'	53-78	55	503	[49]
HPV16 E6 (R)	5'-AGC TGG GTT TCT CTA CGT GTT CT-3'	557-533			
HPV16 E7 (F)	5'-CAA TAA TAT AAG GGG TCG GTG GA-3'	480-500	55	468	
HPV16 E7 (R)	5'-TTT TTC CAC TAC AGC CTC TAC AT-3'	945-923			[49]
β-globin (F)	5'-GAA GAG CCA AGG ACA GGT AC-3'	4-23	55	268	[49]
β-globin (R)	5'-CAA CTT CAT CCA CGT TAC ACC-3'	273-256			
p53 Exon 5 (F)	5'-TAC TCC CCT GCC CTC AAC AA-3'	316-355	55	184	[48]
p53 Exon 5 (R)	5'-CAT CGC TAT CTG AGC AGC GC-3'	499-480			

Briefly, the method involved a 25 μl reaction mix containing 100-200ηg DNA, 10 mM Tris-Cl (pH 8.4), 50 mM KCl, 1.5 mM MgCl_2_, 12.5 μM of each dNTP (dATP, dCTP, dGTP and dTTP), 5 pmoles of each oligonucleotide primer and 0.5 U Taq DNA polymerase (Perkin-Elmer Biosystems, Foster City, CA, USA). The temperature profile used for amplification constituted an initial denaturation at 95°C for 5 min followed by 30 cycles with denaturation at 95°C for 30 sec, annealing at 55°C for 30 sec and extension at 72°C for 30 sec which was extended for 7 min in the final cycle. The HPLC-purified oligonucleotide primers were custom synthesized commercially by M/s Microsynth GmbH (Balgach, Switzerland).

### qRT-PCR

Highly sensitive qRT-PCR was performed for detection of low copy number HPV infection. qRT-PCR employed in the study had the capability of detecting as low as 5 copies of HPV genome per reaction which is equivalent to 0.005 HPV copies per cell equivalent using GP5+/GP6+ consensus primers with Biorad SYBR Green Supermix kit and iCycler PCR (Biorad, Hercules, CA). p53 exon 5 primer sequences (Table 2) were used to normalize the HPV DNA with respect to human genomic DNA. qRT-PCR was calibrated using 10 fold serial dilutions of HPV16 International Standards (IS; ranging from 10^4^-10^1^) supplied under WHO Global HPV LabNet program. Background genomic DNA from HPV-negative cell line C33a was used as negative control and for dilution of standards as prescribed by WHO. Briefly, the amplification was performed in 25 μl reaction mix containing 5 pmol each of GP5+ and GP6+ primers and 30-50ηg of genomic DNA in 5 ul of test volume. The amplification ramp included first step for 3 min at 95°C for initial denaturation, followed by denaturation cycle of 20 sec at 95°C, an annealing cycle of 15 sec at 40°C and an elongation and readout cycle of 30 sec at 72°C for 45 cycles. The specificity was verified by a dissociation curve analysis. Linear plots of the log copy number vs the number of the threshold cycles was consistently obtained for HPV16 L1 amplification and the correlation coefficient was between 0.995 to 1.000 in each run.

## Results

Of 252 patients studied, 62% were in pre-menopausal stage while the remaining 38% formed the post-menopausal group. Clinical staging showed 7.14% (18/252), 14.3% (36/252), 23.01% (58/252), 23.8% (60/252), 26.6% (67/252) and 5.15% (13/252) of the patients belonging to stage I, IIa, IIb, IIIa, IIIb and IV respectively, whereas histopathologic grading revealed 22.60% (57/252), 50.8% (128/252) and 26.6% (67/252) of the tumors in grade I, II and III respectively (Table 3). All the patients were of Indian origin and inhabitants of either New Delhi, North-East India region, southern India, Jammu & Kashmir or Goa region. None of the patients showed family history of any other cancer. DNA isolated from all the cases were employed for detection of HPV infection in biopsy and blood DNA using both conventional as well as highly sensitive real-time PCR methods with pre-calibrated WHO's HPV16 international standards. The tests were able to detect up to 5 HPV genome equivalents per reaction i.e. 0.005 HPV copies per cell equivalent. Human placental DNA or DNA derived from HPV negative C33a cells, HPV18 positive HeLa cells and DNA derived from HPV16 positive cervical cancer biopsies were used as controls.

**Table 3:** Detailed pathological classification and clinical stages of 252 breast cancer patients

Pathological classification	Clinical stages
Infiltrating Ductal Carcinoma (IDC) (218)	Stage I (14)
	Stage II (83)
	Stage III (114)
	Stage IV (7)
	
Mixed IDC & mucinous (20)	Stage I (4)
	Stage II (7)
	Stage III (8)
	Stage IV (1)
	
Medulllary Invasive carcinoma (8) Papillay invasive carcinoma (6)	Stage II (4)
	Stage III (5)
	Stage IV (5)
	

IDC; Infiltrating ductal carcinoma

**Figure 2:**
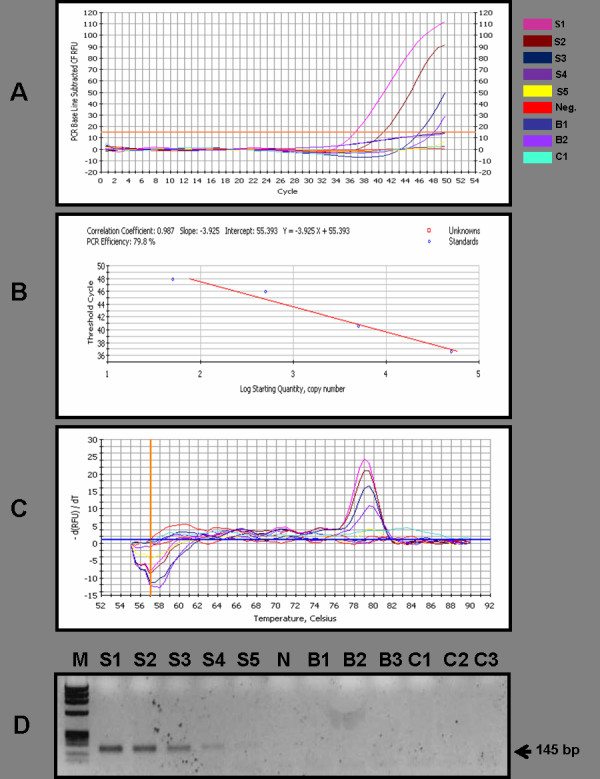
**(A-D) Quantitative Real time PCR of HPV**. **Quantitative Real time PCR of GP5+/GP6+ to detect presence of HPV infection in blood and tumour biopsy DNA from breast cancer patients**. Genomic DNA isolated from blood and tumour tissue biopsies from breast cancer patients were amplified using GP5+/GP6+ L1 consensus PCR primers using Biorad iQ SYBR Green supermix as described in Methods. Ten fold dilution series of WHO HPV16 international standards in the 4 log dynamic range (5 × 104-100) in C33a DNA diluents were used as reference for generation of standard curves. (A) DNA amplification; (B) Standard curve analysis showing efficiency of reaction and calculation of viral copy number; (C) Melt curve analysis showing specificity of HPV16 amplicon and (D) Post real time PCR electrophoresis run of standards and analyzed sample amplicons.

No HPV DNA was detected using consensus primers located within the conserved L1 region of HPV genome by performing 3 to 4 times repeated conventional PCR in any of the 228 breast tumor and 142 blood DNA specimens, while β-globin showed amplification of 268 bp product in all the cases (Figure 1B). The repeat PCR of first PCR products also failed to detect any HPV DNA sequences. When these tumor DNA samples were processed for highly sensitive qRT-PCR, not a single sample was found positive for the presence of HPV DNA (Figure 2A-D). We again checked these samples with type-specific HPV16 E6/E7 primers, but all the samples were found negative for any of HPV infections (Figure 1C &1D).

## Discussion

Despite breast cancer being one of the most frequently diagnosed cancers in women, the etiology and molecular pathobiology of breast carcinogenesis is still not clearly understood. In addition to various exogenous, endogenous, hormonal and genetic risk factors responsible for breast carcinogenesis, its viral etiology particularly implicating HPV remains highly controversial. Although HPV infection, particularly the HR-HPV types 16 and 18, have been strongly implicated in the development of cervical [52] and other cancers [53-63], the evidence for an oncogenic role of HPV in breast cancer is limited.

Our previous study on a limited number of patients [32] as well as several other reports worldwide [27-31] did not observe any infection of HPV in breast cancer, whereas a moderate (20-45%) to high frequency (60-85%) of HPV infection has been reported by many authors [33-42]. Recent reports of detection of very high rate of HPV infection in breast cancer up to 85% [43-46] and in breast cancer cell lines [47] prompted us to re-look into the prevailing condition of HPV infection in breast cancer which we refuted previously [32]. Since in earlier study we used mainly fine needle aspirated (FNAC) cells from breast cancer patients (n = 26) including four tumor biopsy specimens to detect HPV, in the present study we have screened as many as 228 sporadic breast cancer biopsy specimens and 142 blood samples from a total of 252 breast cancer patients with no history of HPV infection or HPV-associated malignancies for the detection of HPV infection. The samples were collected from four different geographic regions of the country and two different PCR techniques both conventional and highly sensitive qRT-PCR were employed. The L1 consensus primers MY09/11 or GP5+/GP6+ and HPV16 E6/E7 (Table 2) which detect as may as 40 mucosal HPV genotypes, including the most prevalent high risk (HPV 16 & 18) and low risk (HPV 6 & 11) types were used but none of the samples by either methods revealed presence of HPV DNA. Additionally, blood samples from 24 familial breast cancer cases which had defined hereditary etiology for development of breast cancer failed to show presence of HPV DNA.

Of 22 previous studies on HPV in breast cancer till date, 15 reported presence of HPV infection in breast cancer and the authors have tried to put forward their possible explanation for detection of HPV DNA in breast cancer. Although the spreading of genital HPV infection from one site to the other is not established, a few studies have reported detection of genital HPVs in peripheral blood lymphocytes of patients with urino-genital cancers [64-67] and head & neck cancers [68]. So, it has been suggested that, there is a possibility of systemic spread of oncogenic virus through organ perfusion [69] facilitating its entry into the breast through circulation. For this reason we tried to look for HPV DNA in blood samples of 142 breast cancer patients from Bangalore, North-east region, J & K and Goa but none of them was found positive for HPV DNA. However, the detection of genital HPVs in the nipple and areolar region reported by de Villers et al. [44] suggests an alternative and perhaps more likely route of infection. Because of varied sexual behavior it is possible that genital HPVs may transmit to breast through oro-genital route or directly through genital-breast contacts. So there could be transmission of HPV infection by mechanical path where the virus is scrubbed through the skin [44]. However, the lack of detection of HPV in breast cancer indicates that the virus has no role to play in breast carcinogenesis in Indian women.

It is also interesting to note that several authors found both positive and negative results from the same region [27,29,43,44]. Studies showing positive as well as negative results for the presence of HPV infection in breast cancer and benign breast tissues are listed in Tables 4 and 5. The reports that show different results from the same country are also indicated. This could be due to variable sexual practices, composition of study subject and different HPV detection methods employed. de Villiers et al [44] reported an extremely high positivity of HPV DNA in 25 out of 29 samples (86%) in invasive breast carcinoma and in 20 out of 29 samples (69%) of the corresponding nipple samples collected from USA. The most prevalent HPV genotypes detected in both benign and breast tumors were HPV11 followed by HPV6 which are low risk HPV-types that generally cause benign lesions and warts and are non-oncogenic. Only 12% cases were found to have infection of high risk HPV type 16 but no HPV18 genotypes could be detected. Bratthauer et al [27], in contrast, analyzed 43 cases of breast cancer from USA for the detection of HPV DNA but surprisingly no HPV infection was observed by these authors in breast cancer cases.

**Table 4:** List of studies identified HPV DNA sequences in breast cancer tissues

S. No.	Study report	Sample size	Total HPV positivity n (%)	HPV16 n (%)	HPV18 n (%)	HPV16/18 n (%)	Other HPV types n (%)	Country
1	Di Lonardo et al., 1992	70	7 (10)	7 (100)	0	0	0	Italy
2	Henning et al., 1999	41	19(46.3)	19 (100)	0	0	0	Sweden
3.	Yu et al., 1999	72	19(26.3)	0	0	0	19 (HPV 33)	China
4.	Yu et al., 2000	32	14(43.8)	0	0	0	14(HPV33)	China
5.	Liu et al., 2004	17	6(35)	3(50)	1(17)	0	0	China
6.	Damin et al., 2004	101	25(24.7)	14(56)	10(40)	1(4)	0	Brazil
7.	Widschwendter et al., 2004	11	7(63.7)	7(100)	0	0	0	Austria*
8.	de Villiers et al., 2005	29	25(86.2)	3(12)	0	0	22(88)	USA*
9.	Kroupis et al., 2006	107	17(15.9)	14(82.3)	0	0	3(17.6)	Greece
10.	Kan et al., 2005	50	24(48)	0	24(100)	0	0	Australia
11.	Gumus et al., 2006	50	37(74)	0	20(54)	0	35(94.6)	Turkey
12.	Khan et al., 2008	124	26(20.9)	26(100)	0	0	0	Japan
13.	de Leon et al., 2009	51	15(29.5)	10(66.7)	3(20)	2(13.3)	0	Mexico
14.	Mendizabal-Ruiz et al., 2009	67	3(4)	1(33.3)	1(33.3)	0	1(33.3)	Mexico
15.	Heng et al., 2009	26 biopsies	8 (30.7)	1 (3.8)	7 (26.9)	0	0	Australia
		9 cell lines	2 (22)	0	2 (22)	0	0	

**Table 5:** List of studies that did not detect HPV DNA sequences in breast cancer tissues

S. No	Study report	Sample size	Total HPV positivity n (%)	HPV16 n (%)	HPV18 n (%)	HPV16/18 n (%)	Other HPV types n (%)	Country
1	Wrede et al., 1992	92	0	0	0	0	0	UK
2	Bratthauer et al., 1992	43	0	0	0	0	0	USA*
3.	Gopalkrishna et al., 1996	30	0	0	0	0	0	India
4.	Czerwenka et al., 1996	20	0	0	0	0	0	Austria*
5.	Lindel et al., 2007	81	0	0	0	0	0	Switzerland
6.	de Cremoux et al., 2008	50	0	0	0	0	0	France
7.	Hedau et al. (Present study)	252*	0	0	0	0	0	India

* Country with both positive and negative results.

Similarly, from Austria, Widschwendter et al [43] analyzed only 11 cases of breast cancer, of which 7 (63.7%) had HPV infection while Czerwenka et al [29] from the same country reported complete absence of HPV infection in breast cancer cases. The reason(s) for disparity of presence or absence of HPV infection in breast cancer cases from the same country is not understood. It may be primarily because of selection of primer types and the amplicon region in the HPV genome which could be the possible explanation for the observed differences in HPV detection. It may also in part, be attributed to cross-contamination during collection and processing of biological samples from other organ sites, if infected with HPV.

Interestingly, in Brazilian women, Damin et al [38] could not observe HPV DNA in benign breast disease, but HPV could be detected in 25% of patients with breast carcinoma. However, HPV infection was found to have no correlation with prognosis of the disease. Lindel and her colleagues [30] showed no evidence of HPV infection in 81 Swiss women with breast carcinomas using the SPF1/2 primers covering about 40 different low, intermediate and high risk HPV types. Similarly, de Cremoux et al [31] analyzed 50 invasive breast carcinoma tissues from French patients using consensus and type-specific primers but no HPV infection were detected in any of breast cancer cases. Wrede et al [28] analyzed a group of 95 British women with breast cancer for the infection of HPV6b, 11, 13, 16, 18, 30, 31, 32, 33, 45 and 51 but they also failed to detect any HPV infection. Kroupis et al [45] performed HPV test on 107 breast carcinomas in Greece and only 17 (15.9%) were found positive. Many of them were having multiple infections of as many as 21 high risk HPV types and majority (14/17; 67%) of them were positive for HPV16. Yu et al [35] analyzed 72 patients from Shanghai, China and Tokushima in Japan found 34.1% positivity for HPV33 alone but there was no evidence of HPV16-positive breast cancer. Yet another study by Mendizabal-Ruiz et al [41] analyzed 67 breast cancer patients including 40 non-malignant tissues and a very low 4.4% (3/67) frequency of HPV infection was observed. Although there exists conflicting reports, it does not insinuate that the reports that did not find viruses in breast cancer specimens were attempting to confirm or refute previous reports but failed to detect HPV. However, it is also clear from these studies that presence of HPV infection in breast cancer may not be a universal phenomenon. The prevalence of HPV infection in breast cancer may differ between places and population and several factors may contribute to this effect. This is amply clear from a recent interesting study of Heng et al [47] who analyzed HPV DNA sequences in as many as nine human breast cancer cell lines including the commonly used MCF-7 cells but only two cell lines (MDA-MB-175 VII and SK-BR-3) were found positive for HPV18. It is, however, not unlikely that these two breast cancer cell lines might have got contaminated in the laboratory with most commonly used HeLa cells that harbor HPV18.

Several other authors from different parts of the world reported presence of HPV infection in breast cancer cases. It is, however, most important to note that not many studies have indicated or analyzed if the women with breast cancer recruited for HPV detection also had anogenital or oropharynheal lesions with HPV infection. Although the route of HPV transmission from one organ to the other is not clear, Hening et al [34] demonstrated HPV16 in 46% of breast carcinoma patients who had a history of CIN III lesions. Most interestingly, all patients with HPV16 positive for breast cancer correspond to same patients with cervical CIN III lesions with HPV16 infection. This is indicative of possible transmission of virus from ano-genital organs to the breast. One of the route discussed earlier could be through blood circulation [64-68]. So, it is essential to look for genital or oropharyngeal infection of HPV in breast cancer patients to rule out the possibility of inter-organ transmission and/or cross contamination of viral infection. However, published data suggest that various epidemiological factors, sexual behavior including the history of HPV-associated diseases specific to the region may play a role in differential distribution of HPV in breast cancer [30].

In spite of exceedingly higher prevalence of genital HPV infection in India as compared to that in Europe and in the USA including presence of other conducive risk factors such as early age marriage, multiple pregnancy, malnutrition, poor hygiene and healthcare, the complete absence of HPV infection in Indian women with breast cancer in the present study does not suggest the role of HPV in breast carcinogenesis in Indian women. It is suggested that although HPV DNA is undetectable, serologic assay may be useful in revealing exposure by detecting presence of antibodies against HPV. But natural HPV infection being poorly immunogenic, the antibody titer will be too low to be detected with the existing serological tests. HPV serology is presently not available in our laboratory and is being developed under the WHO LabNet programme. We however, do not preclude the possibility of yet unidentified HPV playing important role in breast carcinogenesis in India. Furthermore, due to the changing social and psyco-sexual behavior particularly in urban Indian population, it may not be unlikely to find, presence of HPV in breast tissue in future due to an exterior transfer of HPV form genital organs to the breast.

## Conclusions

Complete absence of HPV DNA either in the tumor tissue or in the blood of the same breast cancer patients recruited from four different regions of the country by employing the highly sensitive qRT-PCR does not support the etiological role of oncogenic HPV in the pathogenesis of breast cancer in Indian women.

## Abbreviations

HPV: human papilloma virus; PCR: polymerase chain reaction and Breast carcinoma

## Competing interests

The authors declare that they have no competing interests.

## Authors' contributions

SH - carried out experiments, analyzed the data, and drafted the manuscript. S Hussain, DB, MMM - provided breast cancer biopsy samples, clinical diagnosis and information from J & K. UK, SP, AT, RK - to perform some of the experiments and critical reading of the manuscript. NJ, SS - has done all work related to real time PCR. TD - provided blood samples of breast cancer patients, diagnosis and clinical information from Goa. SC - provided breast cancer biopsy and blood samples, diagnosis and clinical information from Silchar. YMS - provided clinical breast cancer biopsy and blood samples, diagnosis and clinical information from Imphal. ACB - co-designed the study and revised the manuscript. KS - collection of blood and biopsy samples from Bangalore and critical reading of the manuscript. BCD - conceived and designed the study, interpreted the data and critically corrected and communicated the manuscript. All authors have read and approved the final version of the manuscript.

## Pre-publication history

The pre-publication history for this paper can be accessed here:

http://www.biomedcentral.com/1471-2407/11/27/prepub
